# A Rare Case of Pigmented Eccrine Poroma on the Trunk Mimicking Malignant Melanoma

**DOI:** 10.1002/ccr3.70000

**Published:** 2024-12-16

**Authors:** Saman Al‐Zahawi, Alireza Ghanadan, HamidReza Mahmoudi, Zahra Razavi

**Affiliations:** ^1^ Department of Dermatology Razi Hospital, Tehran University of Medical Sciences (TUMS) Tehran Iran; ^2^ Department of Dermatopathology Razi Hospital, Tehran University of Medical Sciences (TUMS) Tehran Iran; ^3^ Autoimmune Bullous Diseases Research Center Razi Hospital, Tehran University of Medical Sciences Tehran Iran

**Keywords:** eccrine glands, malignant melanoma, porocarcinoma, poroma

## Abstract

Representative clinical images in this study can increase awareness regarding the clinical similarity between this benign adnexal tumor and malignant melanoma and highlight the importance of pathological examination.

## Graphical abstract

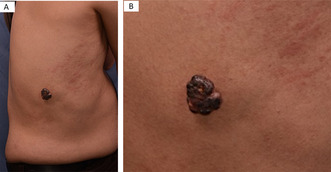

## 1 Case Presentation

A 40‐year‐old male patient presents with a brownish‐black colored mass on the right flank. The mass started as a brownish bump and slowly grew throughout 20 months before presentation to our dermatological center. He did not experience ulceration of the lesion nor did he observe bleeding.

**FIGURE 1:**
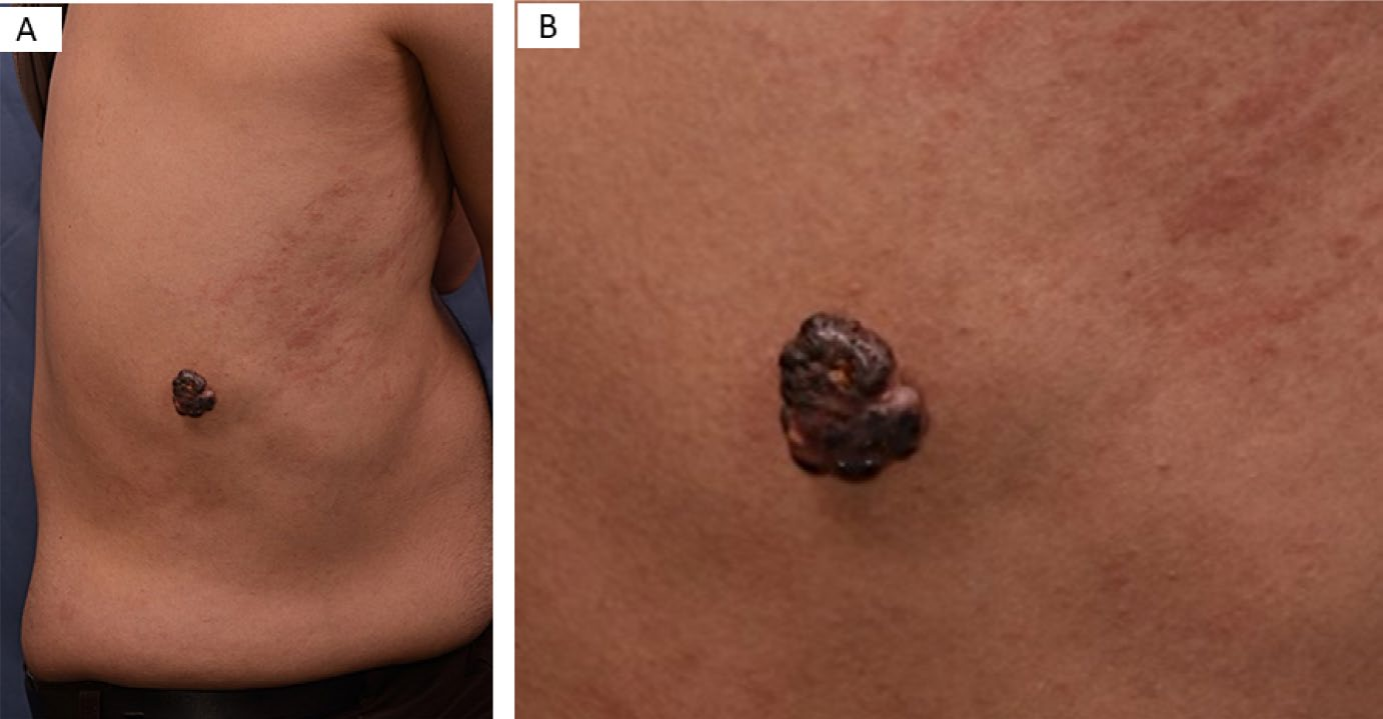
Well‐defined, hyperpigmented nodule in the right flank (A), (B) more close view of the nodule.

On examination, a well‐defined, hyperpigmented nodule, measuring nearly 3.6 × 3.0 × 1.5 cm, with firm consistency was observed (Figure 1). An excisional biopsy with a 2 mm free margin was performed with the differential diagnosis of pigmented melanotic melanoma, irritated seborrheic keratosis, and poroma.

**FIGURE 2:**
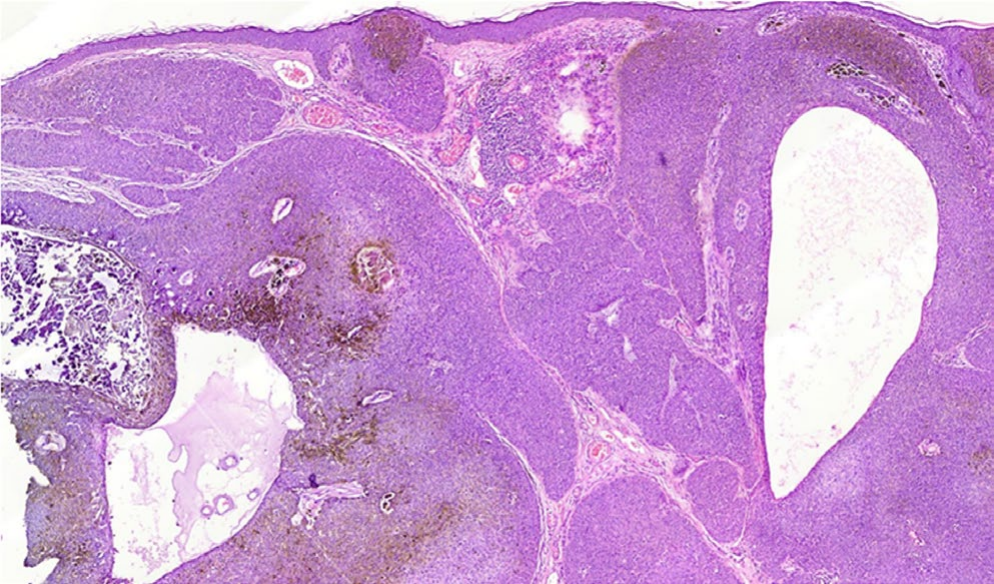
Broad anastomosing bands of small, monomorphic, round poroma cells extending to the epidermis with brown pigmentation and ductal dilatation (40×).

Biopsy reported well‐circumscribing lesion with replacement of the epidermis by the tumoral cells with extension into the dermis in broad anastomosing bands, the bands consisted of small, monomorphic, round poroma cells with brown pigment (Figure 2), and all surgical margins of the lesion were free of tumor. The pathological findings were compatible with Pigmented Eccrine Poroma, which was clinically supported by the benign course of the lesion like slow growth, well‐demarcated lesion, nearly homogenous color.

The patient was followed up for 6 years with no recurrence. Although poroma is a benign adnexal neoplasm, there is a potential for malignant transformation into porocarcinoma. The time for such transformation or triggering factors is unknown, making surgical excision of poroma the gold standard of treatment.

## 2 Teaching Point

Poroma is a rare, benign, adnexal tumor that originates from the terminal duct of the sweat gland. It commonly appears as an erythematous, well‐defined papule‐nodule in the soles or lateral border of the feet; however, 17% of lesions may be hyperpigmented [1]. Few rare cases reported the atypical development of poroma in the trunk with no recurrence after complete surgical excision [2, 3]. When poroma in such an atypical location is pigmented, it might be confused with pigmented tumors such as melanotic melanoma, irritated seborrheic keratosis, or even pigmented basal cell carcinoma. Here we report the rare development of pigmented eccrine poroma in a 40‐year‐old male patient in the right flank.

## Author Contributions

**Saman Al‐Zahawi:** writing – original draft, writing – review and editing. **Alireza Ghanadan:** visualization. **HamidReza Mahmoudi:** conceptualization, supervision. **Zahra Razavi:** conceptualization, supervision.

## Consent

Written informed consent was obtained from the patient to publish this report in accordance with the journal's patient consent policy.

## Conflicts of Interest

The authors declare no conflicts of interest.

## Data Availability

Data are available and it will be provided on request.
